# WhatsApp embedded in routine service delivery for smoking cessation: effects on abstinence rates in a randomized controlled study

**DOI:** 10.1186/s12889-019-6727-z

**Published:** 2019-04-08

**Authors:** Seyfi Durmaz, Isil Ergin, Raika Durusoy, Hur Hassoy, Ayhan Caliskan, Pinar Okyay

**Affiliations:** 1 Ege University Faculty of Medicine, Department of Medical Education, Izmir, Turkey; 2 Adnan Menderes University Faculty of Medicine, Department of Public Health, Aydin, Turkey

**Keywords:** WhatsApp, Abstinence, Smoking cessation, Cessation service, Randomized controlled study

## Abstract

**Background:**

The demand for smoking cessation services has risen in Turkey, as smokers planning to quit reached 35% in 2012. Communication technologies are used globally to support quitters, yet their integration to health services is rare. This study aims to evaluate the effect of support messages through WhatsApp application added to the usual care of a university hospital cessation unit, as compared to usual care alone, on abstinence rates at first month.

**Methods:**

A randomized controlled intervention study was conducted with 132 patients applying to Ege University Hospital’s Department of Public Health Smoking Cessation Clinic, between March and July 2017. Intervention content was prepared and 60 WhatsApp messages about having a plan of action and preventing relapse were developed through expert panels. These messages lasted for 3 months and follow-ups continued for 6 months. The primary outcome was abstinence rate at 1st month post target quit day. As secondary outcomes; the continuous abstinence rates at 3rd and 6th months, number of follow-ups, change in weight and continuity of medication were evaluated. Intention-to-treat analysis was used.

**Results:**

Abstinence rate at 1st month was 65.9% in the intervention group and 40.9% in the control group (*p* = 0.007); 50.0 and 30.7% at 3rd month and 40.9 and 22.7% at 6th month, consecutively (both *p* < 0.05). Being in the intervention group increased abstinence rate by 3.50 (OR, 95% CI = 1.30–9.44) times in the 1st month. When controlled for all other factors in the multivariate logistic regression, the intervention was the only variable significantly associated with abstinence. For secondary outcomes, the intervention increased abstinence rate by 2.50 (OR, 95% CI = 1.08–6.40) times in the 3rd and 2.31 (OR, 95% CI = 1.03–5.16) times in the 6th month. In the intervention group, the number of follow-ups and face-to-face follow-ups were higher at 1st and 3rd months and continuity of medication was longer at 3rd month.

**Conclusions:**

WhatsApp support embedded in cessation service delivery increases the abstinence rate and has favorable effects on follow-up.

**Trial registration:**

This trial is retrospectively registered online at ClinicalTrials.gov with the identifier NCT03714971.

**Electronic supplementary material:**

The online version of this article (10.1186/s12889-019-6727-z) contains supplementary material, which is available to authorized users.

## Background

In Turkey, over 65,300 people are killed annually by tobacco-induced diseases [1]. The high prevalence of consumption in 2016 (30.6%) among adults verifies the tobacco epidemic in Turkey [2]. However, the tendency to give up smoking is increasing [3]. In Turkey, the quitline 171, Tobacco Addiction Treatment Follow-up System (TUBATIS) and Smoking Cessation Outpatient Clinics were established to help those who want to quit smoking. The total number of individuals admitting to cessation services in these clinics was 1,848,462 between 2009 and 2016 [4, 5]. Smokers who want to quit attend these services and get counseling and treatment support for their quit attempt. After a face-to-face counseling session, follow-up is initiated at the first week of quitting [6]. Adjunctions to brief counseling -like telephone counseling- are shown to increase quitting success. [7]. Drug continuity and weight change also play crucial roles throughout the quitting period. Weight gain may even impair the efforts to quit [8]. In some studies it was shown that quitting smoking and weight control efforts interact and improve the success rates for both attempts [9]. Medication, as part of a comprehensive management strategy, is integral to this service with a specified duration prescribed by the physician [10].

Mobile phone applications have facilitated communication by overcoming the difficulties of face-to-face communication. The number of active social media users in the world is 3 billion and social media users via mobile devices comprise 37% of the world’s population [11]. The number of people using WhatsApp, which was 200 thousand at the end of 2013, has quickly risen to 1.3 billion in 2017 [12]. In 2017, 50% of Turkey’s population was using WhatsApp [13].

Studies using mobile technology for smoking cessation and relapse prevention are increasing. Recently, positive results have been obtained from messaging applications like WhatsApp, Facebook and text messages. It was found that smokers preferred to quit by the help of a website, mobile applications, telephone line, e-mail based service, proactive telephone consultation and SMS reminders [14–16]. In 2016, Whittaker and colleagues examined 12 studies with a six-month smoking cessation outcome and found that mobile phone-based interventions increased smoking cessation success by 1.67 (1.46–1.90) [17]. Mobile phone based applications like WhatsApp have also been found to have positive influence on improving knowledge on diabetes or increasing access to mammography screening [18, 19]. In a randomized controlled trial (RCT), the use of WhatsApp and Facebook groups to prevent recurrence after smoking cessation resulted in a statistically significant lower recurrence in both groups compared to the control group [15].

Studies using social media focus on cessation success, pharmacotherapeutic efficacy, evaluation of outpatient services and the opinions of quitters. Such studies are usually carried out in young people and are often not linked to any outpatient services [20–22]. A method embedded in the outpatient service can ease lifestyle changes and strengthen the intervention by the advantage of the technological opportunities. Mobile phone based efforts for quitting tobacco offers the quitter the opportunity to be within reach in any place and in a direct manner, at a time convenient for the user and with low necessity for resources and this reach increases the continuity of motivation throughout the follow-ups. [17].

In this study conducted at Ege University Department of Public Health Smoking Cessation Outpatient Clinic, we tested the hypotheses that; compared to the abstinence rates achieved with usual care, a WhatsApp intervention embedded in the health service with specially designed messages throughout the first 3 months: 1. Increases the abstinence rate at first month (primary outcome), 2. Increases the continuous abstinence rate at third and sixth months (secondary outcomes), 3. Increases the number of follow-ups, continuity to medication and controls weight gain in the first and third months (secondary outcomes).

## Methods

### Trial design

The study was designed as a RCT including two parallel arms: an intervention group receiving messages through WhatsApp Messenger operating on smart phones and a control group not receiving these messages, with an allocation ratio of 1:2. Literature has provided evidence that this allocation does not have a major effect on power [23]. The study was carried out in the smoking cessation clinic of Ege University Medical School’s Public Health Department. Its CONSORT checklist is in Additional file 1.

### Participants

Among patients applying to the smoking cessation outpatient clinic between March and October 2017, > 18-year old volunteers who smoked at least one cigarette/day, who wanted help in quitting smoking, using WhatsApp at least on 4 days of the week and accepting the 3-month follow-up were included in the study. Among a total of 151 patient admissions in the study period, 132 were eligible to be included.

Besides people who did not meet the inclusion criteria, other patients applying to the clinic but who were referred to another center without receiving any treatment, or people who were not ‘ready to quit’ or who had already quit before applying to the clinic were excluded. If two or more patients were living in the same house or had applied to the clinic together, only the first of these was included and the rest were excluded from the study, as they could show the arriving messages to their friend/ partner/ relative.

### Usual care

The usual care provided at the clinic is given by five physicians specially trained on quitting tobacco, working on a daily turn during the week and smokers who admit to the clinic are either given a motivational interview or a quitting counseling, depending on their intention to quit. Those who are ready for quitting are managed through an action plan decided together with the quitter at the first visit and the prevention of relapse is coordinated throughout the follow-ups. In this study, both groups received the standard outpatient care of the clinic including approximately 45 min face-to-face individual counseling at first contact ending with the decision of the treatment and a quit date, and the provision of a support booklet on quitting and subsequent follow-ups by the same clinician at 1st, 2nd, 4th weeks and 3rd month after the quit date, either face-to-face at the clinic or by telephone if the patient does not come to the clinic for follow-up.

### The intervention

The routine service delivery was ongoing as the intervention was conducted. In addition to the routine procedures in service delivery described above, WhatsApp messages were sent to the intervention group according to the plan shown in Table 1.

**Table 1:** The distribution of message content according to topic during the intervention [49]

Main topic	Subtitles	Number of messages^a^
Before the quit day (Having a plan of action)	Controlling the stimulus for smoking	1
	Having a plan of action	2
	Suggesting the healthy behavior	1
	Things to do before the quit day	2
	Individual support from others	1
	Total	7 (Daily)
After the quit day (Preventing relapse)	Importance of self-rewarding	4
	Strategies to cope with nicotine withdrawal	13
	Coping with stimuli for smoking	13
	Changing the environment	10
	Preventing relapse	13
	Total	53 (First month, daily; Second month, every other day; Third month, every 3 days)

^a^Each message may contain more than one topic. The predominant topic has been used for classification

Whatsapp, which enables the transfer and sharing of text, audio, video and documents, is low cost and is becoming more popular with improved encryption. It reduces counseling time in clinical settings [24]. Regardless of the service provider, it enables unlimited use. The sender of the message can track the delivery or read status. Many companies use text messages via SMS for advertisement too often and some people may get exhausted with these texts. As it does not expose the users to advertisements, WhatsApp has become a more preferable choice in personal communication.

### Sample size

An a priori power analysis was conducted with OpenEpi, Version 3 by selecting a two-sided test to compare 30% abstinence rate (ie; the rate calculated for 2016 at Ege University Department of Public Health Smoking Cessation Outpatient Clinic) in the control group at 1st month versus 60% abstinence rate in the intervention group with an error margin of 5%, a power of 80% and an allocation ratio of 1:2 which yielded a minimum sample size of 36 in the intervention and 72 in the control groups [25]. For possible loss to follow-up, the sample sizes were increased by 20% to 43 and 86, respectively.

### Randomization

Among the 132 participants included in the study, 44 were randomly allocated to the intervention arm and 88 were randomly allocated to the control group.

Stratified randomization was achieved by performing a separate randomization procedure within each gender and physician subsets (five physicians at service delivery). Allocation according to gender was conducted regarding the 2:3 female to male ratio in the routine cessation services and stratification according to physician aimed to have a balanced distribution among the different physicians working in the same cessation unit. Randomization was conducted using a computer spreadsheet. The detailed random allocation sequence is presented in Additional file 2.

### Blinding

At the end of their first visit, each patient was sent to the room next-door for recruitment. The patients were detected for eligibility and informed consent was taken. The researcher enrolling participants did not know in advance which treatment the next person would get, which guaranteed allocation concealment. According to the random allocation sequence presented in Additional file 2, patients were allocated to the intervention or control groups. Each patient received an automatic code, which enabled the anonymity of the WhatsApp participant list.

The study was single-blind as blinding of the healthcare providers to intervention assignment was achieved and they conducted their usual care for smoking cessation counseling. The physicians were blind throughout the follow ups as well. However, participants and the researcher who sent the messages were not blind.

### Measures and outcomes

#### Primary outcome: abstinence rate at 1st month

Abstinence rates at the end of the 1st month of follow-up in the intervention and control groups. Abstinence rate at 1st month was calculated with point prevalence. History of cessation was based on self report and those who declared not smoking even a single puff on a cigarette at all in the past 2 weeks were considered as “successful” in the quitting attempt [26].

#### Secondary outcomes

The continuous abstinence rate at 3rd month was defined as not smoking (self report) at all in total in the past 10 weeks and classified as “successful” in the quitting attempt. The continuous abstinence rate at 6th month was defined as not smoking (self report) at all in total in the past 24 weeks, considered as “successful” in the quitting attempt. Another secondary outcome was the total number of follow-ups in the 1st and 3rd months. Contacts were divided into two categories: face-to face contacts and telephone calls. If the total number of routine follow-ups was 3 or more in the 1st month, it was classified as adequate. After the first visit, the first follow-up visit is recommended to be right after the quit day, preferably at the first week. A second follow-up is suggested in the second week and a third follow-up at the fourth week after quit. [6]. This has been basis to our drug continuity measure. At 1st month, at least one of these follow-ups was required to be face-to-face for adequacy. A total of 4 follow-ups was considered as adequate at 3rd month. The continuity to drug/NRT therapy was categorized as < 1 month or ≥ 1 month. At first visit and at first month follow-up, weight was measured in light clothes using a high-quality digital scale. For the 3rd month, self report on weight gain was asked. Any weight change greater than or equal to 1 kg was considered “weight gain”.

#### Control variables

Sex and the physician whom the patient was admitted were controlled.

#### Socio-demographic variables

Sex, age, marital status (married/not married), educational level (secondary school and below, high school and above) social class (unemployed, blue collar, white collar, self-employed) were questioned.

#### Smoking status

Number of cigarettes smoked per day, total pack years, and number of quit attempts were questioned.

#### Medical status

Fagerström Test for Nicotine Dependence has been used to evaluate the level of nicotine dependence [27]. Depression was determined with HADA Scale [28]. CO level was evaluated with the piCO Smokerlyzer carbon monoxide breath test monitor at first visit and at first month of abstinence. Body weight was measured with calibrated equipment which was routinely used at the clinic. Any concomitant disease was asked.

#### Therapy

The quit date, the appropriate therapy type (bupropion, varenicline, nicotine replacement therapy - NRT, or combined as bupropion and NRT), and the physician conducting the counseling were noted. In Turkey, only two forms of NRT are available: nicotine gums and patches.

### Data collection

Data collection was conducted at the Smoking Cessation Clinic of the Public Health Department at Ege University Medical School Hospital between March 2017 and March 2018. The intended number of participants was achieved in 4.5 months and with the completion of the last follow-up, the study was finalized. As part of the routine service delivery, a face-to-face counseling of 45–60 min took place at the first contact, and routine follow-ups were conducted at 1st week, 2nd week; 1st, 3rd and 6th months of quitting. These follow-ups were either by phone call or face-to-face, determined by the patient: At the end of each follow-up, the patients were given the appointment of the next follow-up. If they did not come to the appointment, their physician called them on the phone.

### Development of the message contents

Among 178 key messages gathered through literature review, a selection procedure was led with an expert group. These messages covered evidence based behaviour change techniques for smoking cessation behaviour. The resultant list of messages was tested again with experts and end-users and modified according to the feedback. Similar messages were combined under two main titles: “Having a plan of action preparation” and “Preventing relapse action”. A total of 60 key messages were obtained (Table 1).

Selection procedure of messages: The messages were transformed into graphic images and their timing and frequency were determined. The draft messages were sent via an online questionnaire to seven field experts (four specialists in the fields of public health, pulmonary medicine, medical education & addiction, one primary care physician, one psychologist and one nutritionist) experienced in smoking cessation and seven individuals with quitting experience (seven volunteers- four women and three men-who used WhatsApp more than 4 days a week, had a quitting experience between 1 month and 1 year and who had used cessation services at the same clinic).

Participants were asked to rate each graphic message in terms of content and appropriateness of timing on a Likert-type scale (1: Absolutely no - 5: Absolutely yes) and to add their comments. Average scores were calculated for each graphic message for both expert groups. Messages with an average score of 4.0 and over were considered appropriate and the rest were revised according to the participants’ comments. After the revision, messages were sent back to the expert groups again and they were asked to evaluate with the same scale. Total score averages for 60 messages were 4.94 ± 0.25 for the field experts, 4.65 ± 0.56 for the quitters and, 4.80 ± 0.43 overall. In this round, all messages’ average scores were found above the cut-off point of 4.0. It was concluded that a consensus was established on the graphic messages in terms of the content and timing.

### Analysis

The analyses were conducted according to the Intention-To-Treat (ITT) principle. The participants lost to follow-up were considered unsuccessful in quitting [29].

The groups were compared with the incidences and relative efficacy of the intervention. Chi-square test, Student’s t test in independent groups, single and multivariate logistic regression (enter method) were used for analyses. The variables which were significant in the Chi-square and Student’s t test were put in the multivariate analysis. The multivariate analysis of factors associated with smoking abstinence was performed with two logistic regression models. In the first model we controlled for age and gender, in the second model we controlled for age, gender and all the other associated variables in univariate analyses. Analyses were made separately for the 1st, 3rd and 6th month. Statistical significance was set at *p* < 0.05.

### Ethical issues

The study was approved by Ege University Medical School’s institutional review board (decision no.16–12.1/11 on January 6, 2017). Written informed consent of every participant to be enrolled and to receive WhatsApp messages were also obtained with confidentiality for name and address. With the blinding procedures described above, the delivery of routine service for all participants regardless of the allocation to intervention or control group was ensured.

## Results

Among 151 individuals who applied to the outpatient clinic between March and July 2017, 132 (87.4%) people were included in the study. Three were under 18 years of age, nine were referred to another unit, one was not using a mobile phone, two had a wife/husband enrolled in the study, three had already quit before arriving to the first interview, one was not ready to quit, so these 19 patients were excluded from the study. All of the remaining patients meeting the inclusion criteria volunteered to participate in the study.

**Fig. 1:**
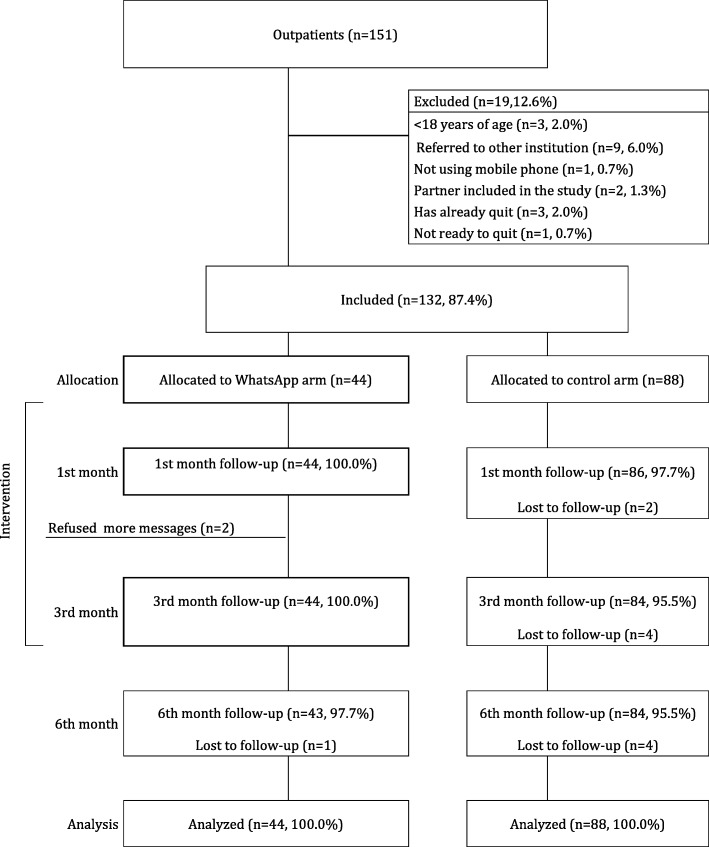
CONSORT flow diagram of the RCT

All of the 44 (100%) participants in the intervention group were followed up at 1st and 3rd month, with only one loss to follow-up at 6th month. Two participants have refused to receive more messages after their 1st month follow-up. The 3rd and 6th month follow-ups of these two individuals were conducted. In the control group, 86 (97.7%) individuals were followed up at the 1st month and 84 (95.5%) at the 3rd and 6th months. Figure 1 shows the flow diagram of the progress through the phases of our RCT consisting of two parallel groups.

Among the participants, 39.4% were female and the mean age of the participants was 39.3 ± 12.1. The other characteristics of participants are shown in Table 2 and Additional file 3.

**Table 2:** Sociodemographic, smoking characteristics and health status of participants (total, intervention, control)^a, b^

	Total		Intervention		Control	
	n	%^a^	n	%^a^	n	%^a^
Gender (*n* = 132)						
Female	52	39.4	16	36.4	36	40.9
Male	80	60.6	28	63.6	52	59.1
Age (*n* = 132)						
18–24	13	9.8	4	9.1	9	10.2
25–34	41	31.1	12	27.3	29	33.0
35–44	32	24.2	12	27.3	20	22.7
45–54	30	22.7	12	27.3	18	20.5
55+	16	12.1	4	9.1	12	13.6
Marital status (*n* = 132)						
Not-married	66	50.0	21	47.7	45	51.1
Married	66	50.0	23	52.3	43	48.9
Education (*n* = 132)						
Secondary school and below	30	22.7	6	13.6	24	27.3
High school and above	102	77.3	38	86.4	64	72.7
Social class (*n* = 117)						
Unemployed	11	9.4	1	2.6	10	12.8
Blue collar	52	44.4	14	35.9	38	48.7
White collar	50	42.7	22	56.4	28	35.9
Self employed	4	3.4	2	5.1	2	2.6
Daily cigarette consumption (*n* = 132)						
≤ 10	14	10.6	2	4.5	12	13.6
11–20	64	48.5	22	50.0	42	47.7
21–30	32	24.2	14	31.8	18	20.5
> 30	22	16.7	6	13.6	16	18.2
Pack-years (*n* = 128)						
≤ 10	39	30.5	14	32.6	25	29.4
11–20	42	32.8	15	34.9	27	31.8
> 20	47	36.7	14	32.6	33	38.8
Quit attempts (*n* = 132)						
Yes	23	17.4	6	13.6	17	19.3
No	109	82.6	38	86.4	71	80.7
Nicotine dependence level (*n* = 132)						
Low	14	10.6	3	6.8	11	12.5
Medium	89	67.4	30	68.2	59	67.0
High	29	22.0	11	25	18	20.5
Depression score (*n* = 130)						
Normal	83	63.8	29	67.4	54	62.1
High	47	36.2	14	32.6	33	37.9
CO value (*n* = 132)						
0–6	13	9.8	4	9.1	9	10.2
7–10	12	9.1	3	6.8	9	10.2
> 10	107	81.1	37	84.1	70	79.5
Concomitant disease (*n* = 132)						
No	31	23.5	9	20.5	22	25
Yes	101	76.5	35	79.5	66	75
Consulting physicians (*n* = 132)						
A	27	20.5	9	20.5	18	20.5
B	19	14.4	9	20.5	10	11.4
C	26	19.7	8	18.2	18	20.5
D	30	22.7	9	20.5	21	23.9
E	30	22.7	9	20.5	21	23.9
Pharmacotherapy (*n* = 131)						
NRT	48	36.6	9	20.9	39	44.3
Bupropion	22	16.8	9	20.9	13	14.8
Varenicline	13	9.9	4	9.3	9	10.2
Combined^b^	48	36.6	21	36.6	27	30.7

^a^Column percent, ^b^ Bupropion + NRT

There was no statistically significant difference between the intervention and control groups in terms of all variables (*p* > 0.05) (Table 2).

**Fig. 2:**
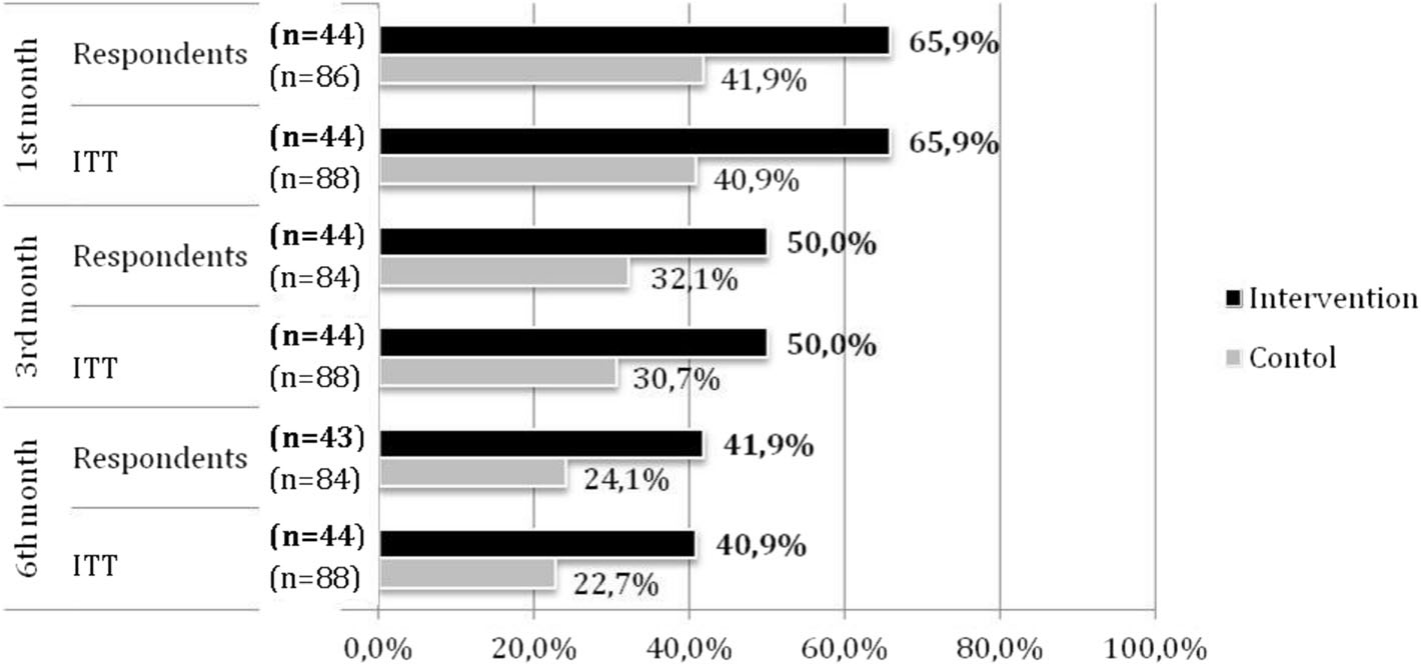
Smoking cessation success rates among respondents, first, third and sixth month follow ups (intervention, control groups)

Of all the individuals included in the study; 49.2, 37.1 and 29.5% successfully quit smoking by the end of the first, third and sixth months, respectively. Abstinence rate at first month was 65.9% in the intervention group and 40.9% in the control group (41.9% among respondents). The continuous abstinence rate in the third month was 50.0% for the intervention group, and 30.7% for the control group (32.1% among respondents). Finally the continuous abstinence rate in the sixth month was 40.9% for the intervention group (41.9% among respondents) and 23.9% for the control group (25.0% among respondents). Quitting success rates among respondents and success rates according to the ITT principle are shown in Fig 2.

The results of the univariate and multivariate logistic regression analyses of the first month follow up are presented in Table 3. At the end of the first month, in Model 1; abstinence rate was 2.85 (OR, 95% CI =1.33–6.14) times higher in the intervention group than the control group. Quitting success among the unemployed was lower compared to white-collar workers (OR = 0.08, 95% Cl = 0.01–0.66). An increase in the depression score was associated with a decrease in abstinence rate (OR = 0.89, 0.80–0.99). With each increase in the number of quit attempts, the abstinence rate increased by 1.39 (OR, 95% CI =1.08–1.79) times. In Model 2, only being in the intervention group was shown to make a statistically significant difference in quitting success by increasing the success probability by 3.51 (OR, 95% CI =1.30–9.44) times.

**Table 3:** Univariate and multivariate logistic regression analysis of factors associated with smoking cessation success in the first and third month follow up^a-f^

		Model 1^a^	Model 2^b, c^
		OR (95% CI)	OR (95% CI)
First month follow up			
Group, n (%)			
Intervention	29 (65.9%)	2.85 (1.33–6.14)^d^	3.51 (1.30–9.44)^d^
Control (ref)	36 (40.9%)	1.00	1.00
Social class, n (%)			
Unemployed	1 (9.1%)	0.08 (0.01–0.66)^d^	0.27 (0.25–2.91)
Blue collar	27 (51.9%)	0.85 (0.38–1.88)	1.07 (0.39–2.90)
White collar (ref)^f^	31 (57.4%)	1.00	1.00
Depression score, mean (±SD)	5.2 (±2.8)	0.89 (0.80–0.99)^d^	0.90 (0.78–1.04)
Quit attempts, mean (±SD)	3.0 (±2.0)	1.39 (1.08–1.79)^d^	1.32 (0.97–1.78)
Third month follow up			
Group, n (%)			
Intervention	22 (50.0%)	2.34 (1.07–5.09)^d^	2.50 (1.04–5.98)^c^
Control (ref)	27 (30.7%)	1.00	1.00
Social class, n (%)			
Unemployed	0 (0.0%)^e^	0.15 (0.02–1.29)^e^	0.42 (0.04–4.23)^e^
Blue collar	25 (48.1%)	1.35 (0.59–3.07)	1.94 (0.77–4.88)
White collar (ref)^f^	22 (40.7%)	1.00	1.00
Depression score, mean (±SD)	4.9 (±2.5)	0.88 (0.78–0.99)^d^	0.89 (0.78–1.02)
Marital status, n (%)			
Married	32 (48.5%)	1.90 (0.82–4.38)	1.61 (0.63–4.12)
Not married (ref)	17 (25.8%)	1.00	1.00
6th month follow up			
Group, n(%)			
Intervention	18 (40.9%)	2.31 (1.03–5.16)^d^	2.31 (1.03–5.16)^d^
Control (ref)	21 (23.9%)	1.00	1.00

^a^Model 1: Adjusted for age and sex,

^b^Model 2: Adjusted for age, sex and other variables

^c^Nagelkerke R^2^ (First month) 0.253; R^2^ (Third month): 0.213 R^2^ (Sixth month):0.126

^d^*p* < 0.05

^e^Agresti adaptation was applied due to the presence of zero in a cell [50]

^f^Two individuals in the ^“^self-employed” class were added to the ^“^white collar” class

In the third month follow up, in Model 1; quitting success of the intervention group was 2.34 (OR, 95% CI =1.07–5.09) times higher than the control group. Each increase in the depression score was associated with a 0.88-fold (OR, 95% CI = 0.78–0.99) decrease in quitting success. The quitting success of men was 2.93 (OR, 95% CI = 1.31–6.57) times higher than women. In model 2, only being in the intervention group was found to be significant in multivariate regression analyses of all variables associated with the third month success. Quitting success within the intervention group increased 2.50 times and gender lost the significance.

At sixth month, being in the intervention group significantly increased success rate by 2.31 (OR, 95% CI =1.03–5.16) times, according to model 1 adjusted for age and gender. Each increase in age was associated with the quitting success increased by 1.04 (OR, 95% CI =1.01–1.08). Age maintained the significance in Model 2.

By the end of the first month, the intervention group was 2.45 times (OR, 95% CI =1.14–5.27) more likely to have adequate follow-up compared to the control group and face-to-face follow-up increased 2.80-fold (OR, 95% CI =1.30–6.04). By the end of the third month, being in the intervention group increased total follow-up adequacy by 3.53 times (95% CI = 1.59–7.82), and face-to-face follow up by 2.62 times (OR, 95% CI =1.21–5.70). While there was no significant difference between groups in terms of continuity to medication by the end of the first month, being in the intervention group in the third month meant 3.33-fold (OR, 95% CI =1.19–9.29) increase in continuity to medication. No significant difference was found between intervention and control groups in terms of weight gain (Table 4).

**Table 4:** Follow-up, continuity to medication and weight gain status of the control and intervention groups by the end of first and third month^a-d^

	Intervention	Control (ref)	OR (95% CI)
	n (%)^b^	n (%)^b^	
First month^a^			
Adequate total follow-up (*n* = 132)	23 (52.3)	29 (33.0)	2.45 (1.14–5.27)^c^
Adequate telephone follow up (*n* = 132)	34 (77.3)	56 (63.6)	1.97 (0.86–4.52)
Adequate face-to-face follow up (*n* = 132)	23 (52.3)	26 (29.5)	2.80 (1.30–6.04)^c^
Medication continuity (*n* = 125)	21 (50.0)	29 (34.9)	1.89 (0.88–4.04)
Weight gain (*n* = 64)^d^	12 (57.1)	20 (55.6)	1.07 (0.39–2.89)
Third month^a^			
Adequate total follow-up (*n* = 132)	22 (50.0)	21 (23.9)	3,53 (1.59–7.82)^c^
Adequate telephone follow up (*n* = 132)	4 (9.1)	7 (8.0)	1,13 (0.31–4.12)
Adequate face-to-face follow up (*n* = 132)	22 (50.0)	26 (29.5)	2,62 (1.21–5.70)^c^
Medication continuity (*n* = 115)	11 (27.5)	8 (10.7)	3,33 (1.19–9.29)^c^
Weight gain (*n* = 64)^d^	15 (53.6)	19 (52.8)	1,03 (0.38–2.78)

^a^Adjusted for age and sex, ^b^Column percent, ^c^*p* < 0.05, ^d^Analysis executed only for quitters

## Discussion

The results of this study showed a benefit of providing additional support via WhatsApp, compared to usual care alone, confirmed at all follow-up points. Regarding the secondary outcomes, the intervention has also increased sufficiency in total follow-ups as well as face-to-face follow-ups. Continuity to medication had increased in the intervention group at 3rd month.

### Determinants of cessation

#### Gender

In the first month of cessation, gender did not have a significant effect on abstinence rates, whereas in the third and sixth month, men were more successful. This finding is similar to previous studies [30–32]. In terms of social support and motivation, the advantageous position of men at home seems to positively contribute to the smoking cessation process [33].

#### Social Class

The unemployed individuals in this study, although not a very representative group, had a lower abstinence rate at first month. Unemployment is found to be associated with very low rates of quitting [34]. Unemployment has the potential to negatively affect many domains of life and financial difficulties can impair the efforts for cessation [35]. In a study conducted in England, the unemployed quitters were less likely to accept aid for smoking cessation compared to other occupations [36].

#### Quit attempts

At first month, as the number of former quit attempts increased, the abstinence rates increased, but after the third month this relationship disappeared. Their previous experience in quitting seems to positively impact their last attempt in the first weeks, but further in the process, they may not have been able to cope with the stimuli that had caused their previous relapses. Previous failures might need to be a more in-depth uncovering to increase awareness and coping strategies. Studies have reported that previous quit attempts interfere with the latter cessation efforts [37] but there are controversial results [38, 39].

#### Depression score

The depressive mood at the time of cessation had effects on abstinence rates at first and third months. Evidence suggests that, the mood of the individual determines cigarette consumption behavior and may also disrupt the efforts for cessation. It has been shown that depression scores are in inverse relation with cessation success [40, 41].

### The role of the intervention

At all points of the follow up, when all the above determinants were controlled in multivariate analysis, the only effective determinant for abstinence was the intervention.

With the intervention the abstinence rates increased 3.5 times at first, 2.5 at third and 2.3 times at sixth month compared with the control group getting only usual care. Although the intensity of the intervention decreased after the first month, the effect on success continued. The meta-analysis (primary outcomes assessed at < 3 months) by Graham and colleagues concluded that interactive internet interventions were 2.10 (OR, 95% CI = 1.25–3.52) times more effective than smoking cessation interventions with printed materials. Internet intervention was found to be 1.35 (OR, 95% CI = 0.97–1.87) times more successful than face-to-face contact but this was statistically insignificant. Twenty-four studies involving different forms of internet interventions had a significant effect of 1.16 fold (OR, 95% CI = 1.03–1.31) in favour of internet interventions [21]. The WhatsApp intervention in our study (for 1st month OR = 3.51, 95% CI = 1.30–9.44; for 3th month OR = 2.50, 95% CI = 1.04–5.98) was found to be even more effective than the interventions in these studies. In a meta-analysis in 2016, Whittaker and colleagues examined 12 cessation studies with outcomes for six-month follow-up. They found that mobile-phone-based interventions increased smoking cessation success rates 1.67 times (OR, 95% CI = 1.46–1.90) [17]. This is in line with our study’s sixth month results (OR = 2.31, 95% CI = 1.03–5.16).

Examples of successful interventions using WhatsApp application for smoking cessation support are increasing. In a RCT conducted in Hong Kong in 2015, the relapse rate for smoking cessation in WhatsApp and Facebook groups in the second and sixth months was found to be lower than in the control group, but the difference was significant only in the WhatsApp group [15]. The researchers explained the success of WhatsApp with its role in social support through group communication. In our study, group communication was not preferred because of the possible intra-group dynamics that could disrupt the process. An incorrect information disseminating from intra-group communication or an individual experiencing a motivational decline could be unpredictable confounders. Thus our attempt was to isolate the intervention as much as possible from other difficult-to-control effects.

One of the strengths of this study was the fact that special attention was given to the timing, intensity and contents of the messages. Moreover, evaluation of the messages by former quitters at the development of the content stage might have helped to fully meet the needs of the quitters. The visualization of messages and the use of a trusted application, such as WhatsApp, which is easy to use and protects privacy, may also have affected the delivery of proper messages.

We did not find an effect of gender, unemployment and depressive mood on abstinence in the final logistic regression. Social media interventions are the new means of health promotion while keeping in mind that if equal internet access is not provided, these interventions could lead to inequalities in health [42]. But as internet is accessible by even low socioeconomic groups, then free online interventions could even reduce health inequalities [43]. Regarding cessation efforts, by reaching those who cannot receive adequate cessation aids/follow-ups, these interventions could add substantially to tobacco control efforts. Information and motivation can reach individuals regardless of place and time. This will remove spatial barriers and time constraints for these groups.

Adequacy of the number of follow-ups was higher in the intervention group at first and third months. This significant difference was consistent for face-to-face interviews while it was not obtained for telephone interviews. One reason may be the increased motivation for face-to-face contact in the intervention group. The follow-ups with telephone interview were proactively initiated by the physician while face-to-face contacts needed more active involvement of the quitter. Throughout the intervention, there were messages which reminded the quitter to reach the physician when needed and some messages emphasizing the importance of follow up. These may have provoked the quitter to keep up with their regular appointments. Another reason for the increase in face-to-face follow-ups in the intervention group may be their increased abstinence rates because those who succeed might feel more proud in attending the service and giving feed-back, while the non-succeeding participants may avoid the issue and thus the visit.

There was no significant difference in terms of weight gain among the quitters between the intervention and control groups. Weight gain is a common problem regarding smoking cessation [44]. Messages for promoting healthy life styles regarding dietary habits and physical activity were amongst the intervention content in this study. However we did not achieve any specific advantage of this content.

Drug continuity is another problem in the cessation period. Quitters generally do not adhere to physicians’ recommendation on the duration of treatment and leave their therapy earlier than intended. The inadequate use of the NRT or drug increases the risk of relapse [45]. In this study, it was seen that the intervention group continued their therapy much better than the control group (50,0% vs 34,9%) but this was not statistically significant in the first month. However, in the third month, drug continuity in the intervention group was significantly better in comparison with the control group (27,5% vs 10,7%). In the messages, the emphasis on continuity to therapy has possibly contributed to this effect. Another possibility is that participants who could not succeed in their quit attempt have stopped taking their medication.

### Limitations

Those admitting to the cessation service have been evaluated, thus the results do not represent population based measures.

Moreover, middle-aged and educated male population dominated this study group. This predominance can be seen in other cessation studies conducted in Turkey at tertiary level services [46, 47].

The individuals in the sample were experienced in using WhatsApp so the findings may not be generalized to smokers who are less likely to use mobile apps.

Social desirability may have biased the self-reports on quitting and quitting had not been confirmed with the CO test for most participants at third and sixth months. Yet studies have shown that self-reported cessation was correlated with biological measures [48].

The control group did not receive any messages, so there may be some subject-expectancy effect. Part of the intervention coincided with Ramadan month (fasting time for Muslims) and this may have interfered with the use of medical therapy during cessation for those who were fasting in their medical therapy period.

Throughout the intervention period, medication has not been subsidized by the social security means. This may have entailed a selection bias regarding admission to service in favor of those who could afford. Admission to tertiary service also has access barriers for those without social security.

Secondary outcomes were not assessed at sixth month, as almost all follow-ups were conducted by phone calls.

## Conclusion

The online support provided through WhatsApp embedded in the routine cessation service delivery increased abstinence rates substantially. The intervention was intensively applied in the first month and then diluted in the subsequent 2 months, however the effect of the intervention continued even at sixth month. When controlled for all other variables, only the intervention was found to be effective in cessation success.

Regarding the secondary outcomes, continuous abstinence at 3rd and 6th month, adequate number of follow-ups (especially face-to-face contacts) and continuity to medication were more achieved in the intervention group.

In the difficult process of quitting, individuals may be supported and motivated with the use of internet technology. Losing quitters from follow-up may also be reduced. E-tools developed to support routine service delivery have the potential to increase abstinence rates.

## Additional files

Additional file 1: CONSORT 2010 checklist of information to include when reporting a randomised trial (PDF 454 kb)

Additional file 2: The detailed random allocation sequence (PDF 292 kb)

Additional file 3: Some characteristics of participants (PDF 223 kb)

## Abbreviations

CONSORT: Consolidated Standards of Reporting Trials
ITT: Intention-To-Treat
NRT: Nicotine Replacement Therapy
RCT: Randomized Controlled Trial
SMS: Short Message Service
TUBATIS: Tobacco Addiction Treatment Follow-up System
