# Supplementary material for: WhatsApp embedded in routine service delivery for smoking cessation: effects on abstinence rates in a randomized controlled study
Source: BMC Public Health. 2019 Apr 8;19:387. doi: 10.1186/s12889-019-6727-z (PMC6454636; doi:10.1186/s12889-019-6727-z)
Supplement: Supplementary file 2 — The detailed random allocation sequence (PDF 292 kb) [file 12889_2019_6727_MOESM2_ESM.pdf]

Additional file 2. The detailed random allocation sequence

Monday				Tuesday				Wednesday				Thursday				Friday			
Rank	Physician	Gender	Group	Rank	Physician	Gender	Group	Rank	Physician	Gender	Group	Rank	Physician	Gender	Group	Rank	Physician	Gender	Group
1	A	F	I	1	B	F	I	1	C	F	K	1	D	F	I	1	E	F	K
2	A	F	K	2	B	F	I	2	C	F	K	2	D	F	K	2	E	F	I
3	A	F	K	3	B	F	I	3	C	F	I	3	D	F	K	3	E	F	K
4	A	F	K	4	B	F	K	4	C	F	K	4	D	F	K	4	E	F	I
5	A	F	K	5	B	F	K	5	C	F	I	5	D	F	I	5	E	F	I
6	A	F	K	6	B	F	K	6	C	F	K	6	D	F	K	6	E	F	K
7	A	F	K	7	B	F	I	7	C	F	K	7	D	F	K	7	E	F	K
8	A	F	I	8	B	F	K	8	C	F	K	8	D	F	I	8	E	F	I
9	A	F	K	9	B	F	K	9	C	F	I	9	D	F	K	9	E	F	K
10	A	F	I	10	B	F	K	10	C	F	K	10	D	F	K	10	E	F	K
11	A	F	I	11	B	F	K	1	C	M	K	1	D	M	I	1	E	M	K
1	A	M	K	1	B	M	K	2	C	M	K	2	D	M	K	2	E	M	K
2	A	M	K	2	B	M	I	3	C	M	K	3	D	M	K	3	E	M	K
3	A	M	I	3	B	M	K	4	C	M	I	4	D	M	K	4	E	M	I
4	A	M	K	4	B	M	K	5	C	M	I	5	D	M	K	5	E	M	K
5	A	M	I	5	B	M	K	6	C	M	K	6	D	M	K	6	E	M	K
6	A	M	K	6	B	M	I	7	C	M	I	7	D	M	K	7	E	M	K
7	A	M	K	7	B	M	K	8	C	M	K	8	D	M	K	8	E	M	I
8	A	M	K	8	B	M	K	9	C	M	I	9	D	M	K	9	E	M	K
9	A	M	K	9	B	M	K	10	C	M	K	10	D	M	I	10	E	M	K
10	A	M	I	10	B	M	K	11	C	M	K	11	D	M	K	11	E	M	K
11	A	M	K	11	B	M	K	12	C	M	K	12	D	M	K	12	E	M	I
12	A	M	K	12	B	M	K	13	C	M	K	13	D	M	I	13	E	M	I
13	A	M	I	13	B	M	I	14	C	M	K	14	D	M	I	14	E	M	K
14	A	M	K	14	B	M	I	15	C	M	K	15	D	M	K	15	E	M	I
15	A	M	K	15	B	M	I	16	C	M	I	16	D	M	I	16	E	M	I
16	A	M	I	16	B	M	K												

Example 1: On Tuesday the first female individual to meet with Dr. B will be distributed to the Intervention (I) group and first male individual to the Control (K) group. Dr. B does not know about the allocation.
Example 2: On Thursday the first individual to meet with Dr. D will be distributed to the Intervention (I) group for both sexes. Dr. D does not know about the allocation.
