# Supplementary material for: WhatsApp embedded in routine service delivery for smoking cessation: effects on abstinence rates in a randomized controlled study
Source: BMC Public Health. 2019 Apr 8;19:387. doi: 10.1186/s12889-019-6727-z (PMC6454636; doi:10.1186/s12889-019-6727-z)
Supplement: Supplementary file 3 — Some characteristics of participants (PDF 223 kb) [file 12889_2019_6727_MOESM3_ESM.pdf]

Additional file 3. Some characteristics of participants

	Mean	Std. Deviation
Age (n=132)	39.27	12.12
Daily cigarette consumption (n=132)	22.58	10.07
Number of quit attempts (n=109)	2.60	1.78
Addiction score (n=132)	5.69	2.24
Depression score (n=130)	5.89	3.67
CO (n=132)	20.87	11.33
